# Prognostic Role of Neutrophil, Monocyte and Platelet to Lymphocyte Ratios in Advanced Ovarian Cancer According to the Time of Debulking Surgery

**DOI:** 10.3390/ijms241411420

**Published:** 2023-07-13

**Authors:** Andrea Plaja, Iris Teruel, Maria Ochoa-de-Olza, Marc Cucurull, Álvaro Javier Arroyo, Beatriz Pardo, Irene Ortiz, Marta Gil-Martin, Josep María Piulats, Helena Pla, Claudia Fina, Anna Carbó, Maria-Pilar Barretina-Ginesta, Sergio Martínez-Román, Elvira Carballas, Andrea González, Anna Esteve, Margarita Romeo

**Affiliations:** 1 Medical Oncology Department, Institut Català d’Oncologia (ICO)-Badalona, Badalona Applied Research Group in Oncology (BARGO), Institut d’Investigació Germans Trias i Pujol (IGTP), 08916 Badalona, Spain; iteruel@iconcologia.net (I.T.); maochoa@iconcologia.net (M.O.-d.-O.); mcucurull@iconcologia.net (M.C.); andreagonzalez@iconcologia.net (A.G.); aesteve@iconcologia.net (A.E.); 2 Medical Oncology Department, Institut Català d’Oncologia (ICO)-L’Hospitalet, Hospital Duran i Reynals, Institut d’Investigació de Bellvitge (IDIBELL), 08908 Barcelona, Spain; arroyo@iconcologia.net (Á.J.A.); bpardo@iconcologia.net (B.P.); ireneortiz@iconcologia.net (I.O.); mgilmartin@iconcologia.net (M.G.-M.); jmpiulats@iconcologia.net (J.M.P.); 3 Medical Oncology Department, Institut Català d’Oncologia (ICO)-Girona, Girona Biomedical Research Institut d’Investigació Biomèdica de Girona (IDIBGi), 17007 Girona, Spain; hpla@iconcologia.net (H.P.); cfina@iconcologia.net (C.F.); acarbo@iconcologia.net (A.C.); mpbarretina@iconcologia.net (M.-P.B.-G.); 4 Obstetrics and Gynecologycal Department, Hospital Germans Trias i Pujol, 08916 Badalona, Spain; smartinezro.germanstrias@gencat.cat (S.M.-R.); ecarballas.germanstrias@gencat.cat (E.C.)

**Keywords:** ovarian cancer, prognostic, neutrophil to lymphocyte ratio, monocyte-to-lymphocyte ratio, platelet-to-lymphocyte ratio, type of surgery, inflammatory biomarkers

## Abstract

Despite a multimodal radical treatment, mortality of advanced epithelial ovarian cancer (AEOC) remains high. Host-related factors, such as systemic inflammatory response and its interplay with the immune system, remain underexplored. We hypothesized that the prognostic impact of this response could vary between patients undergoing primary debulking surgery (PDS) and those undergoing interval debulking surgery (IDS). Therefore, we evaluated the outcomes of two surgical groups of newly diagnosed AEOC patients according to the neutrophil, monocyte and platelet to lymphocyte ratios (NLR, MLR, PLR), taking median ratio values as cutoffs. In the PDS group (*n* = 61), low NLR and PLR subgroups showed significantly better overall survival (not reached (NR) vs. 72.7 months, 95% confidence interval [CI]: 40.9–95.2, *p* = 0.019; and NR vs. 56.1 months, 95% CI: 40.9–95.2, *p* = 0.004, respectively) than those with high values. Similar results were observed in progression free survival. NLR and PLR-high values resulted in negative prognostic factors, adjusting for residual disease, *BRCA1/2* status and stage (HR 2.48, 95% CI: 1.03–5.99, *p* = 0.043, and HR 2.91, 95% CI: 1.11–7.64, *p* = 0.03, respectively). In the IDS group (*n* = 85), ratios were not significant prognostic factors. We conclude that NLR and PLR may have prognostic value in the PDS setting, but none in IDS, suggesting that time of surgery can modulate the prognostic impact of baseline complete blood count (CBC).

## 1. Introduction

Epithelial ovarian cancer (EOC) is the leading cause of death from gynecologic cancer in the United States and Europe [1]. Unfortunately, most cases are still diagnosed at advanced stages, such as International Federation of Obstetricians and Gynecologists (FIGO) stage III or IV, presenting a 5-year survival rate of approximately 50%, which is achieved combining an aggressive cytoreductive debulking surgery with platinum-based chemotherapy (mainly carboplatin plus paclitaxel) [2]. Recently, a positive shift in the lethality curves have been observed due to the progressive centralization of surgeries in high-volume centers, and the introduction of PARP inhibitors as maintenance therapy in the first line setting for the vast majority of patients in high-income countries [3]. However, there is still much room for improvement, and a new generation of phase III trials in this setting are testing the role of adding checkpoint inhibitors to current standard-of-care (NCT03737643, NCT03740165, NCT03522246, NCT03602859) [4,5,6,7,8,9].

High-grade serous carcinoma is the most frequent histology (above 80%), and main classical prognostic factors are FIGO stage, surgical outcomes (no residual disease (R0) versus residual disease) and *BRCA1/2* genes mutational status (those mutated being associated to better outcomes and higher platinum-sensitivity) [10,11,12]. Host-related factors, such as advanced age, ECOG > 1 and poor nutritional status at diagnosis, are associated with worse outcomes [13]. Among host-related factors, it is well known that the systemic inflammatory response plays an important role in cancer development and progression, as well as in immune surveillance, both well-established cancer hallmarks that directly impact on cancer prognosis and response to oncologic therapy [14,15], but the best biomarker has not been identified yet.

Many surrogate markers of inflammation and immune surveillance, which could be easily and repeatedly determined in blood analysis, have been studied as prognostic factors in ovarian cancer, including albumin, fibrinogen [16], C-reactive protein [17,18], platelet, leukocyte count and leukocyte subpopulations (neutrophils, monocytes and lymphocytes). There is a body of retrospective evidence of the prognostic significance of the neutrophil–lymphocyte ratio (NLR) [19,20,21,22], monocyte–lymphocyte ratio (MLR) [23,24,25,26,27] and platelet–lymphocyte ratio (PLR) [28,29] in EOC cancer, as well as in several tumor types [30,31]. These inflammatory markers evaluate the potential systemic balance between myeloid-dependent pro-tumor inflammation and lymphocyte-dependent anti-tumor immune response [32]. Overall, high ratios seem to represent an immunosuppressive profile and are associated with worse overall prognosis and aggressivity, being the NLR the most informative ratio [33,34]. However, the small sample sizes and variability in study designs, the potential influence of non-identified confounding variables and the fact that most studies were conducted on the Chinese population, limit the consistency of this statement and, therefore, the generalized utilization of these ratios. The few studies performed on Caucasian populations have shown more modest and controversial results [35,36,37,38,39].

Specifically, it is unknown whether the prognostic role of these ratios may vary according to surgical timings. In advanced EOC, the decision to perform a primary debulking surgery (PDS) followed by chemotherapy or an interval debulking surgery (IDS) after neoadjuvant chemotherapy (NACT) is frequently a subject of controversy. Despite two randomized phase III trials showing non-inferiority of IDS with respect to PDS in terms of overall survival (OS) [40,41], in real-world daily practice, this decision is normally based on a radiological and, when possible, a laparoscopic assessment of the likelihood of achieving an R0 surgery at diagnosis, also balancing the estimated associated morbidity and baseline functional status of the patient [42,43,44]. Nowadays, it is generally accepted that patients who have undergone IDS represent a group with higher risk of relapse or early progression. Additionally, high tumor burden has been associated with local and systemic changes to the immune system and with an immunosuppressive tumor microenvironment (TME) [45]. Considering that patients that are candidates for IDS initially remain for a longer period of time under the influence of higher volume disease, we hypothesized that surgical timings could modulate the importance of blood count-based ratios.

The aim of this study was to evaluate the prognostic implication of baseline NLR, MLR and PLR in two groups of Caucasian advanced EOC, according to the type of surgery performed, whether upfront or interval, and adjusting for *BRCA* status.

## 2. Results

### 2.1. Patients’ Characteristics

**Figure 1:**
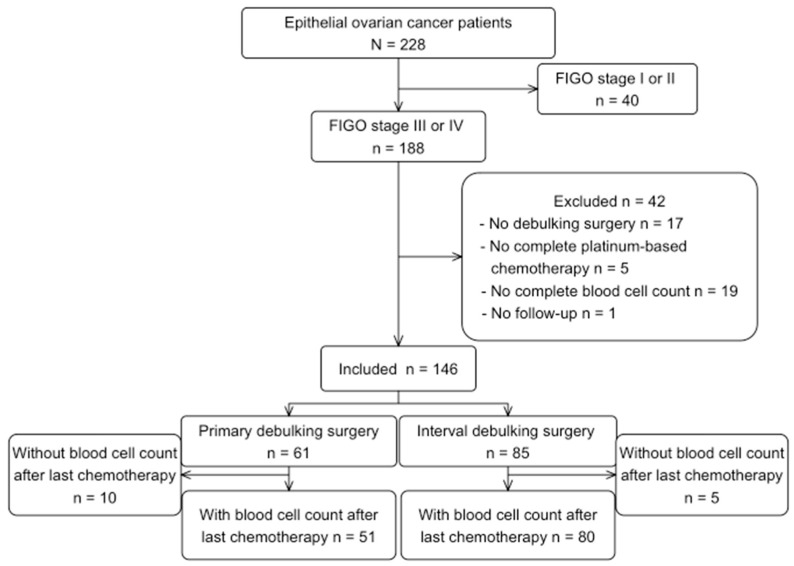
Flowchart of patients’ selection.

From 228 advanced EOC patients initially identified, 146 patients were included for the main analysis. Reasons for excluding patients are shown in Figure 1. The post-treatment analysis included 131 patients, where 15 patients were excluded due to lack of blood samples in the pre-defined time period, or due to neutropenia/thrombocytopenia > G1 Common Terminology Criteria for Adverse Events (CTCAE).

Baseline characteristics and median cut-off of the three ratios in the overall population and each surgical group are shown in Table 1. Overall, median age at diagnosis was 62 years (IQR 55.2-70) and high-grade serous carcinoma was the most frequent histologic subtype (84.9%). Clinical stage was stage III in 101 (69.2%) patients and stage IV in 45 (30.8%) patients. *BRCA* status was known in 114 (78.1%) patients, of whom 30 (20.35%) were *BRCA* mutated. Regarding surgery results, 117 patients (80.1%) had R0, and 29 patients (19.9%) R1/R2.

Primary debulking surgery was performed in 61 women (41.8%), whereas 85 (58.2%) underwent IDS. Main clinical prognostic factors, namely stage, FIGO stage, *BRCA* status and residual disease, did not significantly differ between the PDS and IDS groups. Contrary, median age (58 vs. 65 years, respectively, *p* = 0.0013), percentage of patients with high-grade serous carcinoma (73.8% vs. 92.9%, respectively, *p* = 0.003), and median CA-125 levels (436 vs. 970 UI/mL, respectively, *p* = 0.011) showed statistically significant differences between the two groups. Regarding baseline complete blood count (CBC), median baseline ratios significantly differed between both groups, with higher ratios in the IDS group (*p* = 0.004, *p* = 0.009 and *p* = 0.001 for NLR, MLR and PLR, respectively).

With a median follow-up of 46.9 months (min 6.8–max 126.2), 79.9% of patients had progressed and 58.4% were dead at the time of data analyses. Both groups showed great differences in survival outcomes. Median OS of the PDS and IDS groups was 86.4 months (95% CI: 72.7–not reached (NR)) and 39.8 months (95% CI: 33.8–49.3), respectively (log-rank *p* = 0.0001), and median progression free survival (PFS) was 30.7 months (95% CI: 22–40.8) and 16.4 months (95% CI: 14.8–19.2), respectively (log-rank *p* = 0.00024) (Supplementary Material Figure S1).

### 2.2. Association of Baseline CBC Ratios on Patients’ Outcomes in the PDS and IDS Groups

**Figure 2:**
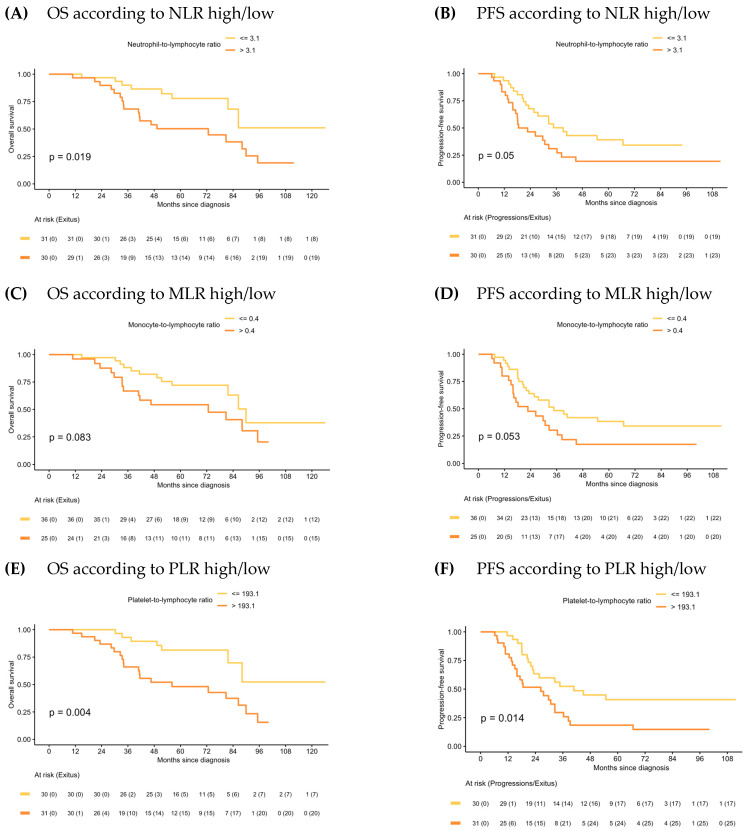
Kaplan–Meier of OS and PFS according to NLR, MLR and PLR in PDS.

In the PDS group, median OS for patients with NLR-low and PLR-low values were significantly higher than for those with the NLR-high and PLR-high values (NR vs. 72.7 months, 95% CI: 40.9–95.2, *p* = 0.019; and NR vs. 56.1 months, 95% CI: 40.9–95.2, *p* = 0.004, respectively). Although there was a trend towards better outcomes among MLR-low patients, OS differences with MLR-high patients were not significant. Similar results were observed in PFS (Figure 2).

**Figure 3:**
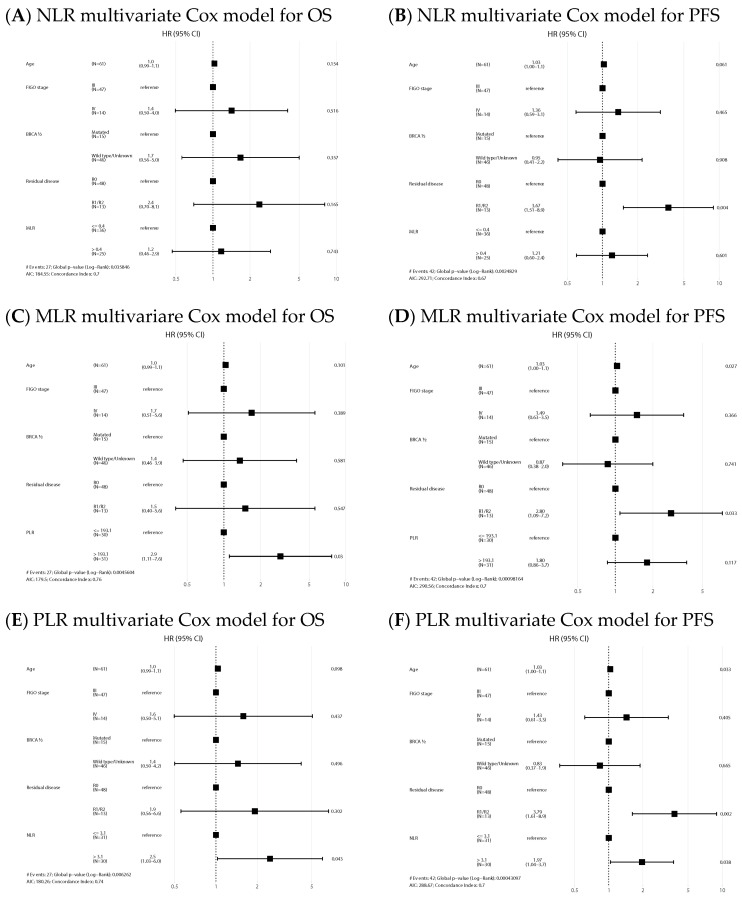
Forest plots of multivariate Cox models for the risk of death (**A**,**C**,**E**) and the combined event progression/death (**B**,**D**,**F**) of each ratio in PDS.

NLR-high and PLR-high values emerged as negative prognostic factors of OS both in the univariate (HR 2.63, 95% CI: 1.14–6.06, *p* = 0.024, and HR 3.32, 95% CI: 1.40–7.89, *p* = 0.007, respectively) and multivariate analysis (HR 2.48, 95% CI: 1.03–5.99, *p* = 0.043, and HR 2.91, 95% CI: 1.11–7.64, *p* = 0.03, respectively). Regarding PFS, NLR and PLR also emerged as negative prognostic factors in the univariate analysis (HR 1.82, 95% CI: 0.99–3.36, *p* = 0.054, and HR 2.13, 95% CI: 1.15–3.96, *p* = 0.017, respectively), but only NLR remained as an independent factor in the multivariate analysis (HR 1.97, 95% CI: 1.04–3.72, *p* = 0.038). Univariate analysis is shown in Supplementary Material Table S1. Forest plots of multivariate Cox models for the risk of death and the combined event progression/death of each ratio in PDS are shown in Figure 3.

**Figure 4:**
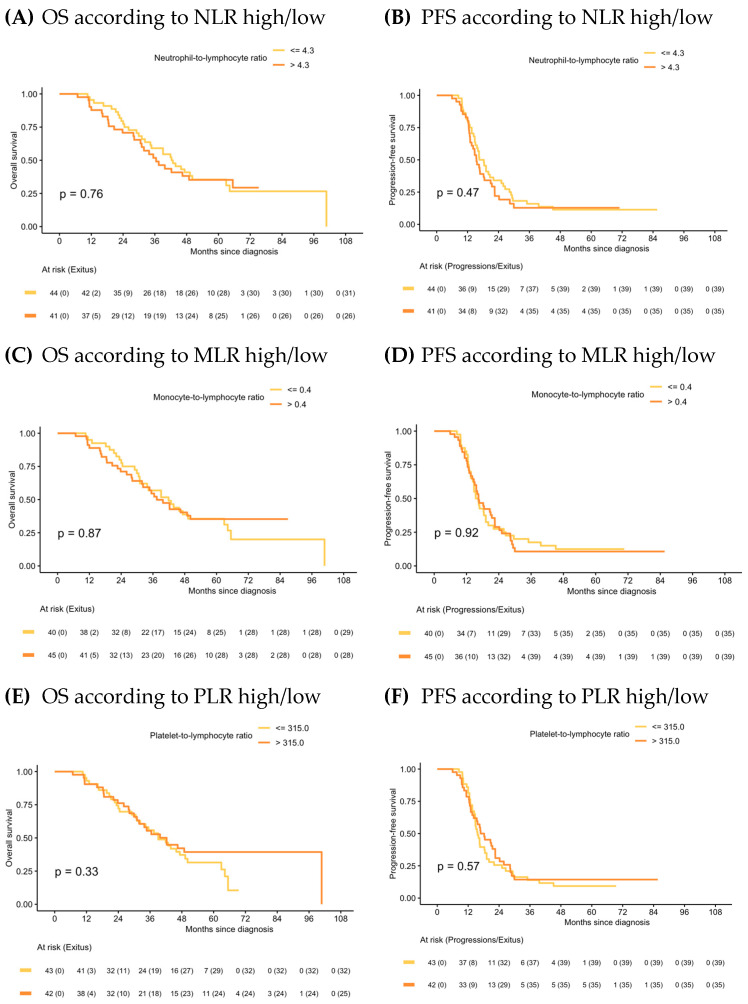
Kaplan–Meier of OS and PFS according to NLR, MLR and PLR in IDS.

**Figure 5:**
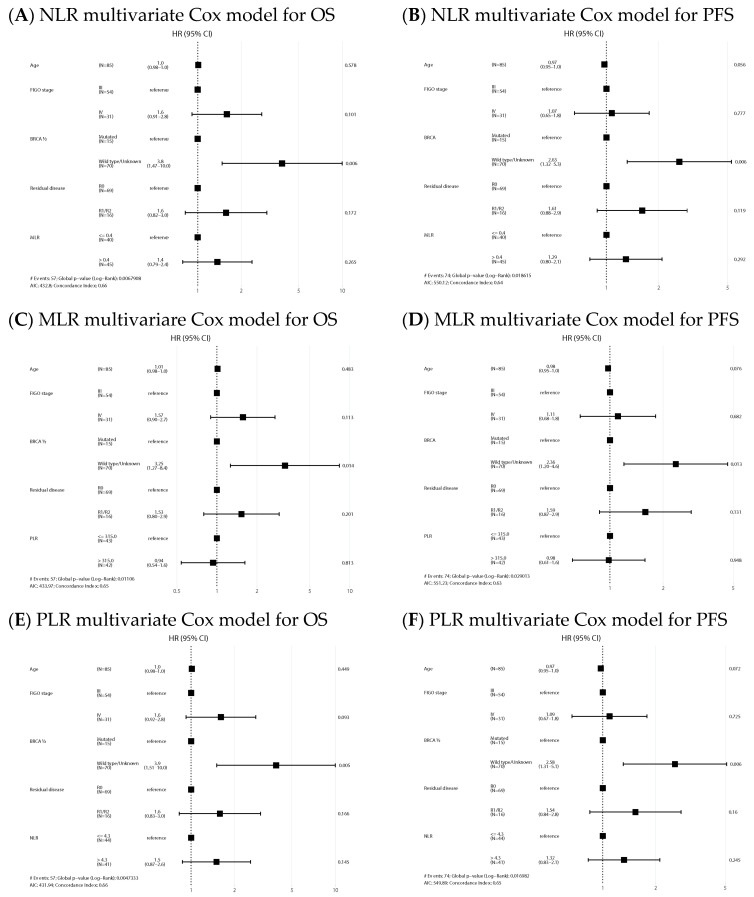
Forest plots of multivariate Cox models for the risk of death (**A**,**C**,**E**) and the combined event progression/death (**B**,**D**,**F**) of each ratio in IDS.

In the IDS group, median OS and PFS in the NLR high/low, MLR high/low and PLR high/low were similar (Figure 4) and ratios were not statistically significant prognostic factors in the Cox models for any of the survival outcomes. Univariate analysis is shown in Supplementary Material Table S2. Forest plots of multivariate Cox models for the risk of death and the combined event progression/death of each ratio in IDS are shown in Figure 5.

### 2.3. Post-Treatment CBC Ratios

Finally, 131 out of 146 patients were included in the post-treatment ratio analysis (61% IDS, 39% PDS). Median post-treatment ratios values were statistically significantly lower than those pre-treatment in the IDS group, and also for NLR and PLR in the PDS group (*p* < 0.001) (Supplementary Material Table S3). Outcomes did not differ significantly between patients with decreased or increased post-treatment ratios, either in the PDS or IDS groups (Supplementary Material Figures S2 and S3).

## 3. Discussion

In this study, we found that baseline high-NLR and high-PLR were independent poor/negative prognostic factors of outcome in EOC patients undergoing primary debulking surgery, but not in patients undergoing interval debulking surgery.

Cancer development and progression is the result of an interplay between tumor cells and host cells [46]. Tumor infiltrating immune cells present in TME can induce a tumorigenic or anti-tumor effect, promoting or hindering angiogenesis, tumor growth, invasion and metastasis [47]. The presence of tumor-infiltrating lymphocytes (TILs) in the TME of ovarian cancer patients has been related with increased OS [48], and myeloid cells, such as neutrophils [49] and macrophages [50], have shown to have either immune-suppressive or anti-tumor effects depending on the cell state, highlighting the importance of deep TME characterization to improve biomarker selection. In this context, previous studies in ovarian cancer have reported that a high infiltration of TILs [48] and tumor-associated macrophages (TAMs) type 0/1 were associated with favorable prognosis, while high infiltration of tumor-associated neutrophils (TANs) were associated with worse outcome [51]. At a systemic level, in peripheral blood, neutrophils have diverse and complex functions through the release of immunosuppressive mediators favoring cancer progression, invasion, angiogenesis and metastasis [52,53,54,55]. Moreover, platelets have been shown to induce epithelial–mesenchymal transition promoting metastasis [56,57], while monocytes are involved in phagocytosis, antigen presentation, and migrate to tissues differentiating into macrophages [58]. Finally, lymphocytes exert a critical role in cancer immune response by directly having a cytotoxic effect on tumor cells [59]. Indeed, a high neutrophil, monocyte and platelet count in peripheral blood has been associated with poor prognosis in several tumors [34,60,61,62], while a low lymphocyte count has been associated with reduced anti-tumor responses and is frequently observed in advanced cancer patients [63]. However, there is no established correlation between these peripheral cell counts and TILs in the TME.

In this context, CBC-based ratios, such as NLR, MLR and PLR, have driven the focus of research in this field. An important number of systematic reviews and meta-analyses have been conducted to assess the prognostic value of immune-based biomarkers in peripheral blood of EOC patients [19,20,21,26,27,29]. Recently, an umbrella systematic review including 17 meta-analyses of retrospective studies demonstrated that baseline high NLR, MLR and PLR were independent predictors of poor OS and PFS [33]. Notably, nearly all studies were carried out among Chinese populations, with some common limitations, such as their retrospective design, limited sample size and, particularly, the influence of confounding variables [64]. The few studies performed in Caucasian populations have shown more modest and controversial results [32,35,36,37,38,39,65,66]. Our research focuses on a Spanish population (mainly Caucasian) and examines the predictive significance of *BRCA* status in multivariate analyses. These aspects serve as strengths of the present study, as they have been underexplored in previous investigations. Our results are consistent with previous studies and suggest that baseline NLR and PLR could be a reliable prognostic biomarker in Caucasian patients with advanced EOC, even after adjusting for *BRCA1/2* status. Additionally, we found an overall decrease in ratios after treatment, suggesting that chemotherapy and surgical removal of the tumor have a significant effect on circulating immune cells [67], despite that the small sample size of our post-treatment analysis (in the PDS and IDS subgroups) may have hampered reaching further conclusions on outcomes.

Interestingly, regarding the setting of surgery performed, we found that these ratios are prognostic factors for OS and PFS only in those patients undergoing PDS, but not in the IDS group. Prior research has mainly focused on patients that underwent PDS, and few studies evaluated patients treated with NACT followed by IDS. Three retrospective studies analyzed the prognostic role of NLR in this context, and only one of them found that high NLR was an independent prognostic factor for worse OS [37,67,68]. Interestingly, a prospective study showed that baseline NLR was not associated with NACT response, but a decrease in the NLR after chemotherapy was correlated with a better response and PFS [69]. To our knowledge, there is only one previous study that analyzed the association between these CBC-based biomarkers and outcomes of advanced EOC according to type of surgery performed. In contrast with our results, they found that high-PLR was independently associated with poor OS in IDS, but not in PDS, and they did not find an association with NLR [70]. Two alternative or even additional reasons could explain our results. On the one hand, the intrinsic worse prognosis of the IDS patients in our study may have masked the power of the ratios as a prognostic factor. This worse prognosis does not seem to be explained by classical prognostic factors, which do not differ significantly among the PDS and IDS groups, but probably by older age and initial unresectability, potential confounding factors that lead to the decision of beginning treatment with NACT. Remarkably, baseline CBC ratios were statistically higher among patients in the IDS group than those in the PDS. On the other hand, our results could also be explained by prior research suggesting that a high tumoral burden promotes an immunosuppressive microenvironment. A persistent high tumor burden (due to the delay in surgery) could lessen the prognostic role of baseline ratios in IDS patients. Globally, our results would highlight the importance of timely-defined surgical subgroups for the analysis of the upcoming results of several phase III trials testing the addition of immunotherapy to the current standard-of-care in first line.

Finally, the predictive role of CBC-based ratios for response to treatment remains to be elucidated. Some studies have already identified cut-off values of NRL and PLR above which platinum-resistance (defined as relapses within the first six months of last platinum dose) can be predicted [71,72,73]. On the other hand, despite there being a strong association between the presence of certain immune cells and angiogenesis, none of these ratios have demonstrated to be a predictive biomarker of response to bevacizumab [32,72]. Additionally, a small study with 20 patients in the recurrent setting suggested that the NLPN score (recurrent NLR × number of previous regimens) could be an independent predictor of olaparib (PARP inhibitor) efficacy in platinum-sensitive patients [74]. However, analyzing the role of CBC-based ratios would be of higher interest in the context of treatment with immunotherapy. In fact, a high-NLR has been significantly associated with worse outcome in patients with 16 different types of cancer treated with immune checkpoints inhibitors (ICI), but these results were not observed in those with endometrial and ovarian cancers [75]. Notwithstanding, high-NLR has been associated with early discontinuation (before 8 weeks) of ICI in monotherapy in EOC [76]. Moreover, an association between these ratios and TILs has been proposed [77], which is of special interest since TILs have shown to have a prognostic and predictive role in response to ICI [78]. As previously mentioned, the scientific community is eagerly awaiting the results of several phase three trials testing the addition of ICI to current standard-of-care maintenance. Thus, developing predictive biomarkers of response to immunotherapy is currently a major priority in ovarian cancer [79,80,81].

We acknowledge that our study has limitations. Firstly, we have performed a retrospective study and the sample size is relatively small. Secondly, the value of CBC-based ratios as a surrogate marker of an anti-tumor immune response is limited by their intrinsic variability and is not only influenced by the TME, but also easily affected by several confounding factors, such as infectious processes, chemotherapy treatments, and surgical complications. However, our cut-off values based on median values of each ratio are aligned with those previously reported in the literature: the cut-off values of NLR reported in ovarian cancer range from 2.11 to 6, with a median of 3.24 [19,20,21,22,28,29]; MLR ranged between 0.23 and 0.54, with a median of 0.26 [23,24,25,26,27]; and PLR ranged between 62.3 and 300, with a median of 205.4 [28,29]. We acknowledge that the proper cut-off of each ratio remains unknown [64], and some studies alternatively chose the cut-off based on receiver operating characteristic (ROC) curve analysis. We explored different methods and decided to use median value, as it was the most consistent cut-off in our cohort and with those reported in previous studies.

## 4. Materials and Methods

### 4.1. Study Design

The target population was composed of newly diagnosed advanced EOC who underwent surgery at three Catalan Institute of Oncology (ICO) sites before 2016, in the pre-IPARP inhibitors era. The ICO is a high-volume cancer monographic institution that provides specialized management of ovarian cancer with unified clinical protocols. Patients were identified from each retrospective clinical databases (ICO-L’Hospitalet between 2011 and 2016, ICO-Badalona between 2008 and 2016, and ICO-Girona between 2013 and 2016 [L’Hospitalet de Llobregat, Badalona and Girona, respectively, Spain]).

Selection criteria were based on histologically or cytologically documented primary invasive EOC, FIGO stage III or IV, having undergone radical treatment based on standard debulking surgery (either upfront or interval) and platinum-based adjuvant/first-line chemotherapy. The inclusion criteria related to systemic treatment required that patients should have received at least four cycles of postoperative platinum-based chemotherapy for patients in the PDS group or a minimum of three neoadjuvant cycles for patients in the IDS group. Moreover, patients should have had available complete blood cell counts (CBC) performed within the four weeks prior to first treatment (either surgery or NACT) to be included in this study.

Baseline patient characteristics retrieved from medical records included age at diagnosis, histologic differentiation subtype, FIGO stage, *BRCA1/2* mutational status (pathogenic mutation vs. not), CA-125 at diagnosis, type of surgery (PDS or IDS), residual disease at surgery (R0 or R1/R2) and chemotherapy treatment (schema, number of cycles and dates). CBC (specifically neutrophils, lymphocytes, monocytes, and platelets) were assessed within 4 weeks prior to first treatment in order to calculate baseline NLR, MLR and PLR, which were defined as the neutrophil, monocyte or platelet count divided by the lymphocyte count, respectively. In addition, we collected the first determined CBC between 1 and 3 months after the last cycle of first-line chemotherapy when no G2 or higher leukopenia or thrombocytopenia was present (CTCAE was accepted) [82], in order to calculate post-treatment ratios. The dates of progression/relapse and death were updated on 21 January 2020.

### 4.2. Endpoints

Main endpoints were the analysis of PFS and OS. PFS was defined as the time from the date of diagnoses until the date of progression/relapse after primary treatment or death for any cause, or last follow-up. OS was defined as the time from the date of diagnoses to the date of death, or last follow-up.

### 4.3. Statistical Analysis

Descriptive statistics were used for demographic and clinical data. Continuous variables were analyzed using median and interquartile range (IQR). Categorical data were reported as frequencies and percentages and compared using chi-square or Fisher’s exact test, as appropriate.

Survival endpoints were estimated using the Kaplan–Meier method and curves were compared between strata using the log-rank test. Baseline NLR, PLR and MLR were categorized as NLR-high/NLR-low, MLR-high/MLR-low and PLR-high/PLR-low, respectively, considering median values in each subgroup of patients (PDS and IDS) as cut-offs. For the exploratory analysis of the effect of increase/decrease in baseline versus post-treatment CBC ratios on survival outcomes, differences were normalized using the baseline ones.

Univariate Cox regression models were used to identify the association of potential prognostic factors with the hazard of death and the combined event, progression/death. Moreover, adjusted HR were estimated using multivariate Cox models including the main known prognostic factors: age, FIGO stage, residual disease and *BRCA* status. Hazard ratios (HRs) and 95% confidence intervals (CIs) were reported.

Statistical analysis was performed using the R software v. 4.1.2.

## 5. Conclusions

In conclusion, we found that NLR-high and PLR-high are independent prognostic inflammatory biomarkers of poor outcomes in Caucasian patients with newly diagnosed advanced epithelial ovarian cancer treated with PDS. These ratios can be readily acquired prior to surgery and provide information about prognostic outcome and aggressiveness of the disease in this subgroup of patients. However, we could not find this prognostic role in the IDS subgroup, suggesting that time of surgery can modulate the prognostic impact of baseline CBC. Further studies analyzing the association between these inflammatory peripheral blood cells, cytokines and the tumor-associated immune cells in surgical samples could provide great information to guide more personalized treatments.

## Figures and Tables

**Table 1:** Baseline clinical and pathological characteristics in the overall population and each surgical group (IDS and PDS).

Clinical–Pathological Characteristics (n,%)	Total N = 146	Interval Debulking Surgery N = 85 (58.2%)	Primary Debulking Surgery N = 61 (41.8%)	*p*-Value
Age: median [IQR]	62.0 [55.2; 70.0]	65.0 [58.0; 73.0]	58.0 [51.0; 65.0]	0.001
≤59 years	57 (39)	23 (27.1)	34 (55.7)	
>59 years	89 (61)	62 (72.9)	27 (44.3)	
Histologic subtype				0.003
High-grade serous	124 (84.9)	79 (92.9)	45 (73.8)	
Others	22 (15.1)	6 (7.1)	16 (26.2)	
*BRCA* ½ status				0.414
Mutated	30 (20.5)	15 (17.6)	15 (24.6)	
Wild type/Un-known	116 (79.5)	70 (82.4)	46 (75.4)	
FIGO Stage				0.118
III	101 (69.2)	54 (63.5)	47 (77.0)	
IV	45 (30.8)	31 (36.5)	14 (23)	
Residual disease				0.872
R0	117 (80.1)	69 (81.2)	48 (78.7)	
R1/R2	29 (19.9)	16 (18.8)	13 (21.3)	
CA-125 (U/mL): median [IQR]	714.4 [262.6; 1756.0]	970.0 [410.8; 2082.2]	436.8 [147.0; 1331.7]	0.011 *
Neutrophil (×10^3^/µL): median [IQR]	5.15 [4.0; 6.5]	5.2 [4.1; 6.6]	4.8 [3.8; 6]	0.165 *
Monocyte(×10^3^/µL): median [IQR]	0.6 [0.5; 0.7]	0.6 [0.5; 0.8]	0.6 [0.5; 0.6]	0.046 *
Platelet (×10^3^/µL): median [IQR]	328 [265.5; 425.5]	367 [279; 468]	303 [253; 361]	0.003 *
Lymphocyte (×10^3^/µL): median [IQR]	1.35 [1.1; 1.8]	1.2 [1.0; 1.6]	1.57 [1.2; 2]	0.004 *
NLR: median [IQR]	3.8 [2.4; 5.3]	4.3 [2.9; 6.4]	3.1 [1.9; 4.7]	0.004
MLR: median [IQR]	0.4 [0.3; 0.6]	0.4 [0.3; 0.7]	0.4 [0.2; 0.5]	0.009
PLR: median [IQR]	258.6 [154.2; 391.1]	315.0 [193.2; 436.4]	193.1 [140.7; 300.0]	0.001
Median OS: months (95% CI)	50.15 (42.2–81.7)	39.8 (33.8–49.3)	86.4 (72.7–NR)	0.0001
Median PFS: months (95% CI)	18.86 (16.8–22.5)	16.4 (14.8–19.2)	30.7 (22–40.8)	0.0004

NLR: neutrophil-to-lymphocyte ratio; MLR: monocyte-to-lymphocyte ratio; PLR: platelet-to-lymphocyte ratio; PFI: platinum-free interval; IQR: interquartile range; OS: overall survival; PFS: progression free-survival; CI: confidence interval. * *p*-value belongs to the non-parametric Mann–Whitney *U* test used to assess if distributions represented by medians of the different blood cell types are equal or not between the two groups.

## Data Availability

The data presented in this study are available in the article and Supplementary Material.

## Supplementary Materials

The supporting information can be downloaded at: https://www.mdpi.com/article/10.3390/ijms241411420/s1.
