# Supplementary material for: Prognostic Role of Neutrophil, Monocyte and Platelet to Lymphocyte Ratios in Advanced Ovarian Cancer According to the Time of Debulking Surgery
Source: Int J Mol Sci. 2023 Jul 13;24(14):11420. doi: 10.3390/ijms241411420 (PMC10380459; doi:10.3390/ijms241411420)

Supplementary Material

Figure S1: Kaplan Meier of overall survival (OS) and progression-free survival (PFS) according the type of surgery performed, primary debulking surgery (PDS) or interval debulking surgery (IDS).

A) OS according type of surgery performed

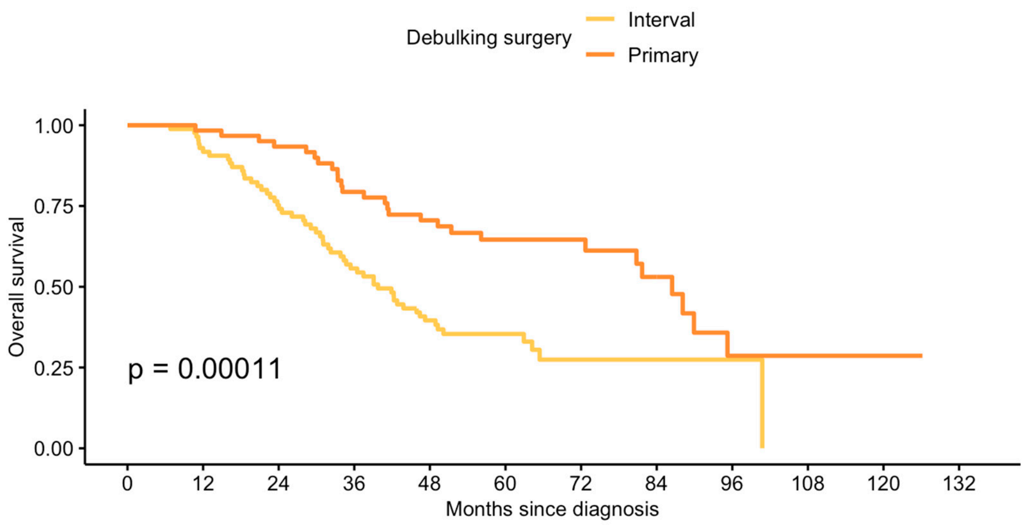

At risk (Exitus)

	0	12	24	36	48	60	72	84	96	108	120	132
Interval	85 (0)	79 (7)	64 (21)	45 (37)	31 (50)	18 (53)	4 (56)	3 (56)	1 (56)	0 (57)	0 (57)	0 (57)
Primary	61 (0)	60 (1)	56 (4)	45 (12)	40 (17)	28 (20)	20 (20)	12 (23)	3 (27)	2 (27)	1 (27)	0 (27)

B) PFS according type of surgery performed

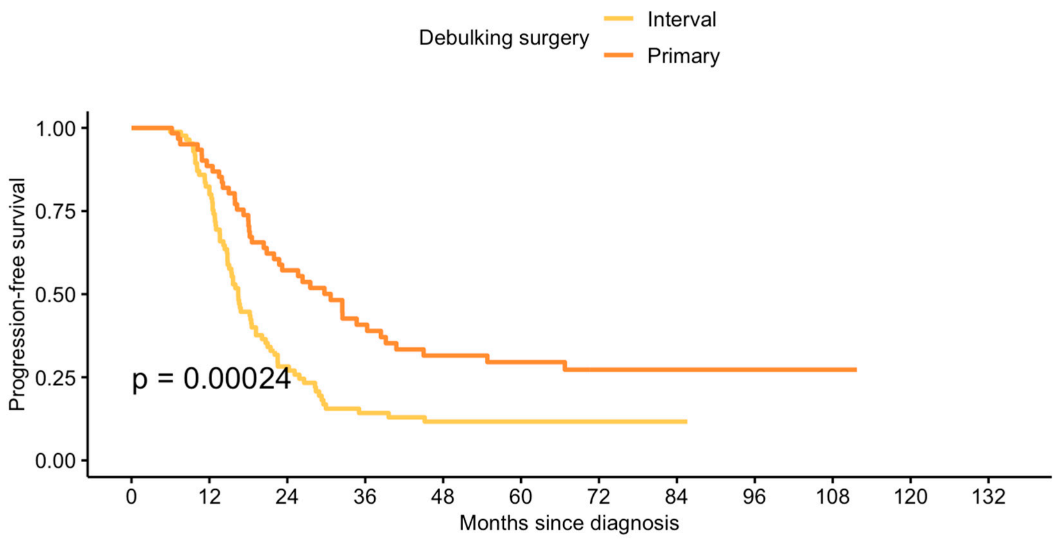

At risk (Progressions/Exitus)

	0	12	24	36	48	60	72	84	96	108	120	132
Interval	85 (0)	70 (17)	24 (61)	11 (72)	9 (74)	6 (74)	1 (74)	1 (74)	0 (74)	0 (74)	0 (74)	0 (74)
Primary	61 (0)	54 (7)	34 (26)	22 (35)	17 (40)	14 (41)	10 (42)	7 (42)	2 (42)	1 (42)	0 (42)	0 (42)

Table S1: Univariate Cox models for the risk of death, and the combined event progression/death, in the PDS subgroup.

	Univariate analysis for OS		Univariate analysis for PFS	
**Variables**	**HR (95% CI)**	**p-value**	**HR (95% CI)**	**p-value**
Age (years)	1.04 (1.00-1.08)	0.047	1.02 (1.00-1.05)	0.089
FIGO stage		0.045		0.014
III	1		1	
IV	2.29 (1.02-5.12)		2.36 (1.19-4.69)	
BRCA ½		0.117		0.381
Mutated	1		1	
WT / Unknown	2.21 (0.82-5.95)		1.39 (0.66-2.91)	
Residual disease		0.006		0.001
R0	1		1	
R1/2	3.33 (1.40-7.93)		4.04 (2.02-8.09)	
NLR		0.024		0.054
≤ 3.1	1		1	
> 3.1	2.63 (1.14-6.06)		1.82 (0.99-3.36)	
MLR		0.089		0.057
≤ 0.4	1		1	
> 0.4	1.94 (0.90-4.17)		1.80 (0.98-3.31)	
PLR		0.007		0.017
≤ 193.1	1		1	
> 193.1	3.32 (1.40-7.89)		2.13 (1.15-3.96)	

OS: overall survival; PFS: progression free survival; NLR: neutrophil-to-lymphocyte ratio; MLR: monocyte-to-lymphocyte ratio; PLR: platelet-to-lymphocyte-ratio; HR: hazard ratio.

Table S2: Univariate Cox models for the risk of death, and the combined event progression/death, in the IDS subgroup.

	Univariate analysis for OS		Univariate analysis for PFS	
**Variables**	**HR (95% CI)**	**p-value**	**HR (95% CI)**	**p-value**
Age (years)	1.01 (0.98-1.04)	0.613	0.98 (0.95-1.01)	0.137
FIGO stage		0.048		0.314
III	1		1	
IV	1.71 (1.01-2.91)		1.27 (0.80-2.04)	
BRCA ½		0.008		0.021
Mutated	1		1	
WT / Unknown	3.50 (1.39-8.78)		2.13 (1.12-4.07)	
Residual disease		0.153		0.117
R0	1		1	
R1/2	1.58 (0.84-2.95)		1.58 (0.89-2.80)	
NLR		0.760		0.473
≤ 4.3	1		1	
> 4.3	1.09 (0.64-1.84)		1.18 (0.75-1.87)	
MLR		0.867		0.921
≤ 0.4	1		1	

> 0.4	0.96 (0.57-1.62)		1.02 (0.65-1.62)	
PLR<br>≤ 315<br>> 315	 1<br>0.77 (0.45-1.30)	0.326	 1<br>0.87 (0.55-1.38)	0.561

OS: overall survival; PFS: progression free survival; NLR: neutrophil-to-lymphocyte ratio; MLR: monocyte-to-lymphocyte ratio; PLR: platelet-to-lymphocyte-ratio; HR: hazard ratio.

Table S3: Median baseline and post-treatment NLR, MLR and PLR.

**PDS<br>N = 51 (39%)**	**Baseline**	**Post-treatment**	**p-valor**
NLR: median [IQR]	3.1 [2.1;4.1]	1.7 [1.3;2.2]	p < 0.001
MLR: median [IQR]	0.4 [0.3;0.5]	0.3 [0.3;0.4]	p=0.112
PLR: median [IQR]	211.2 [147.0;296.2]	147.5 [111.3;192.0]	p < 0.001
**IDS<br>N = 80 (61%)**	**Baseline**	**Post-treatment**	**p-valor**
NLR: median [IQR]	4.3 [2.9;6.3]	1.6 [1.2;2.4]	p < 0.001
MLR: median [IQR]	0.5 [0.3;0.7]	0.3 [0.2;0.4]	p < 0.001
PLR: median [IQR]	315.4 [202.6;437.7]	138.2 [94.7;177.4]	p < 0.001

PDS: primary debulking surgery; IDS: interval debulking surgery; NLR: neutrophil-to-lymphocyte ratio; MLR: monocyte-to-lymphocyte ratio; PLR: platelet-to-lymphocyte-ratio; IQR: interquartile range.

Figure S2: Kaplan Meier of OS and PFS according to increase/decrease of NLR, MLR and PLR in PDS.

A) OS according NLR high/low

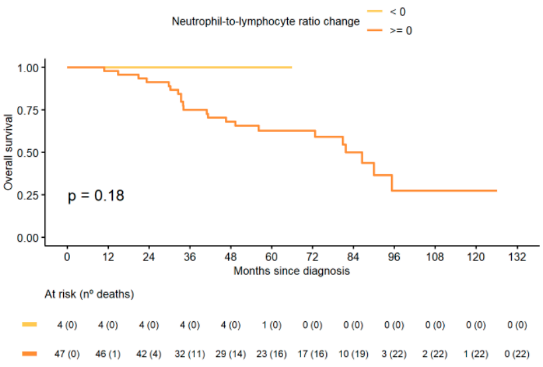

B) PFS according NLR high/low

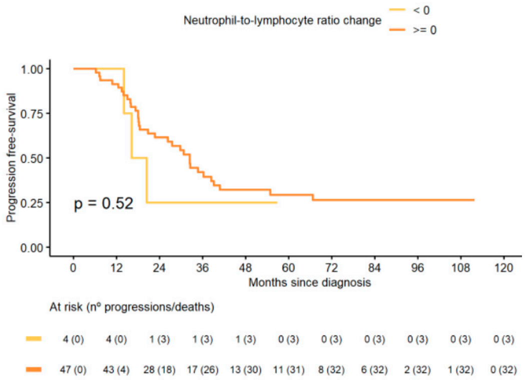

C) OS according MLR high/low

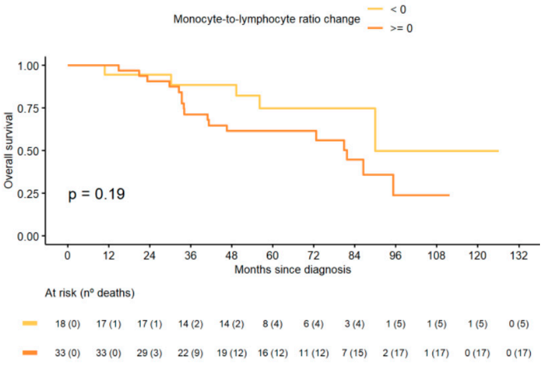

D) PFS according MLR high/low

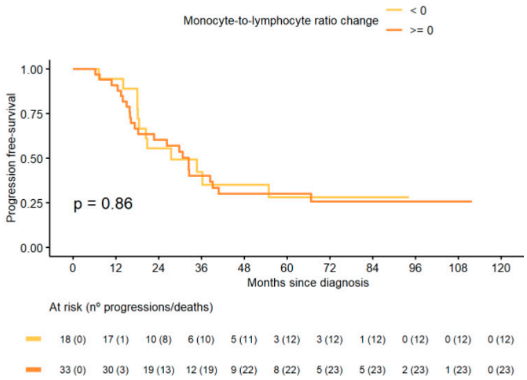

E) OS according PLR high/low

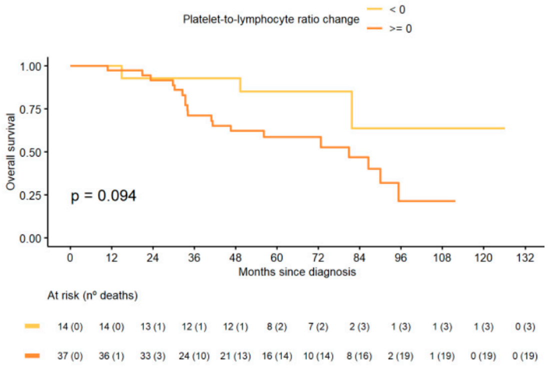

F) PFS according PLR high/low

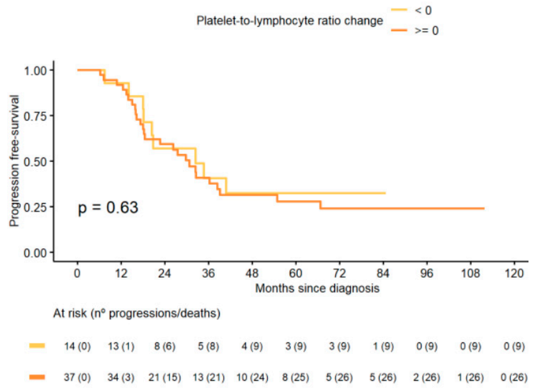

Figure S3: Kaplan Meier of OS and PFS according to increase/decrease of NLR, MLR and PLR in IDS.

A) OS according NLR high/low

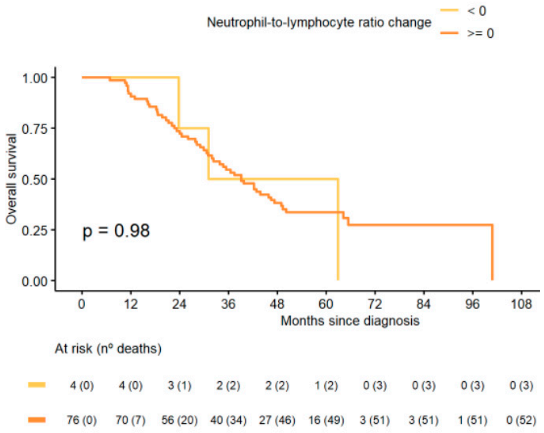

B) PFS according NLR high/low

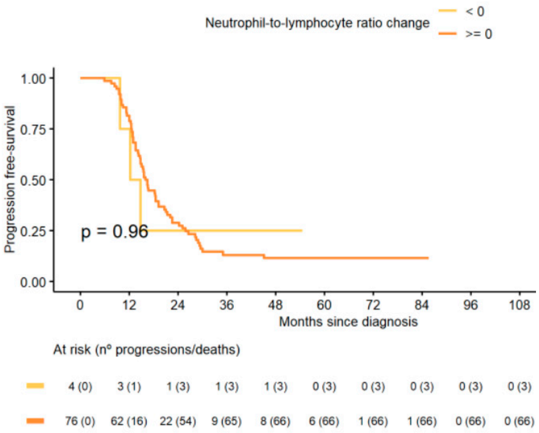

C) OS according MLR high/low

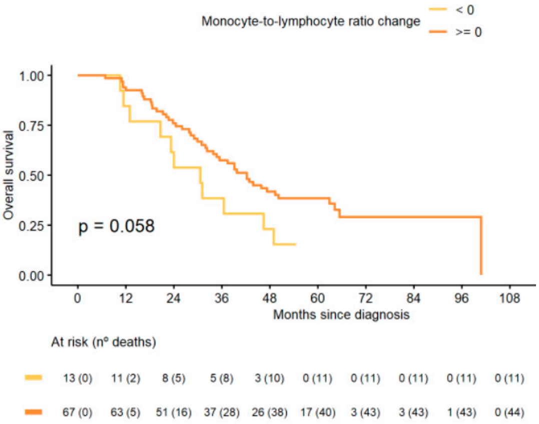

D) PFS according MLR high/low

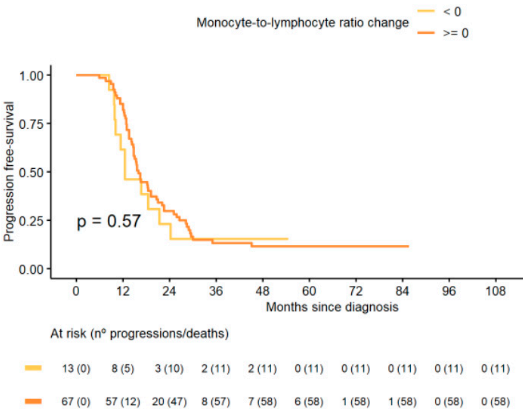

E) OS according PLR high/low

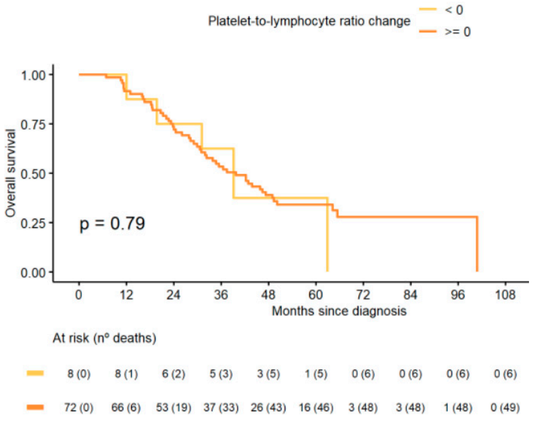

F) PFS according PLR high/low

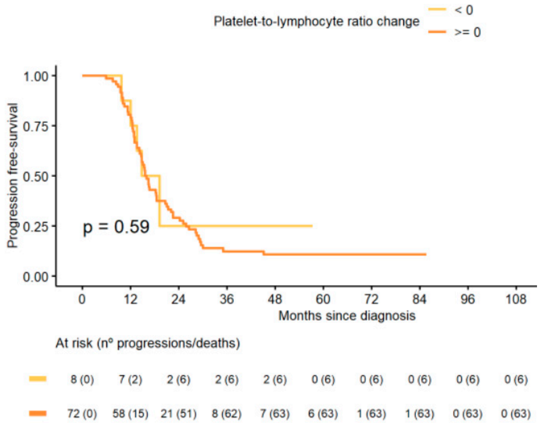

Supplement: Supplementary file 1 [file ijms-24-11420-s001.zip › ijms-2461996-supplementary.pdf]
